# Female loggerhead sea turtles (*Caretta caretta* L.) rarely remate during nesting season

**DOI:** 10.1002/ece3.5869

**Published:** 2019-12-20

**Authors:** Jacob A. Lasala, Colin Hughes, Jeanette Wyneken

**Affiliations:** ^1^ Florida Atlantic University Boca Raton FL USA; ^2^ Mote Marine Laboratory Sarasota FL USA

**Keywords:** breeding sex ratio, mating behavior, paternity analysis, sea turtle

## Abstract

The goal of this study was to assess the consequences of single versus multiple paternity by identifying paternity of clutches per female to identify whether there were detectable costs or benefits. Multiple mating can occur when the benefits of mating outweigh the costs, but if costs and benefits are equal, no pattern is expected. Previous research on loggerhead sea turtle (*Caretta caretta*) populations found male‐biased breeding sex ratios and multiple mating by many females nesting in southwestern Florida. A sample of nesting loggerhead females who laid more than one nest over the course of the season and a subset of their hatchlings were examined from 36 clutches in 2016 on Sanibel Island, Florida. Males that fathered hatchlings in the first clutch sampled were identified in subsequent clutches. Interestingly, 75% of the females analyzed had mated singly. No male was represented in more than one female's clutches. The results suggest that females likely mate at the beginning of the season and use stored sperm for multiple clutches. Evidence for mating between laying events was limited. There was no consistent pattern across the subsequent multiple paternity clutches, suggesting benefits to loggerhead females likely equal their costs and subsequent mating is likely determined by female preference.

## Graphical abstract

The focus of this paper is to examine and describe the costs and benefits of multiple mating across taxa and then focus on a case study by examining repeating nesting of loggerhead sea turtles (*Caretta caretta*) nesting in the Gulf of Mexico. We identified in all nests, males that were present in the first nest were also present in subsequent nests (confirming sperm storage). We also found instances of possible sperm depletion and weak evidence for remating events, but no evidence of male genotypes in multiple female's nests (polygyny).

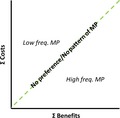

## 1 INTRODUCTION

One of the major components of a mating system is the number of mates an individual mates with within a breeding season (Emlen & Oring, 1977). Multiple mating can lead to multiple paternity (Birkhead & Møller, 1998; Holt & Lloyd, 2010). The number of mates has been extensively studied across variety of taxa and mating systems described; examples include invertebrates (Gotoh, Dansho, Dobata, Ikeshita, & Ito, 2017; Laidlaw & Page, 1984; Pardo, Riveros, Fuentes, Rojas‐Hernández, & Veliz, 2016), birds (Gibbs et al., 1990; Goymann, Makomba, Urasa, & Schwabl, 2015), fish (Masonjones & Lewis, 2000), mammals (Herr & Rosell, 2004), and reptiles (Bollmer, Irwin, Rieder, & Parker, 1999).

Within a breeding season, the number of mates per individual depends on the availability of potential mates, sum of the benefits and costs, and constraints on behavior. Males benefit directly from multiple mating by increasing the likelihood of offspring production, thus increasing individual fitness (Fincke, 1984; Fox & Rauter, 2003; Holman, 2016; Trivers, 1972). Males suffer costs in energy spent competing for females (Dubuc, Ruiz‐Lambides, & Widdig, 2014; Searcy & Yasukawa, 1989), guarding female mates to decrease the risk of sperm competition (Ramm et al., 2015), and in reduction of foraging opportunities (Komdeur, 2001). When sperm production is seasonally elevated, the increase may be energetically costly (Hosken, 2001), although in many species this cost appears to be negligible (Uller & Olsson, 2008). In extreme examples, males can be injured or health may suffer during mating and while competing for mates (Franck et al., 2002; Leonard, Pearse, & Harper, 2002; Nessler, Uhl, & Schneider, 2009). The benefit of increased fitness typically outweighs the costs of multiple mating for males, though males may be constrained by factors such as the availability of receptive females (Andersson, 1994; Birkhead & Møller, 1998).

Females can benefit from multiple mating directly, or indirectly through benefits to their offspring. Direct benefits are often taxon‐specific and can include the transfer of accessory substances within ejaculate, which can stimulate egg maturation (Eberhard & Cordero, 1995; Klowden, 1999), material benefit, such as nuptial food that is consumed by the female (Vahed, 1998), and sufficient sperm for fertilization (Sheldon, 1994). In some taxa, benefits enhance offspring survival such as assistance offspring care (Reding, 2015; Reynolds, 1996; Smith, 1995), or protection against predators (Arnqvist, 1989; Pardo et al., 2016). Several indirect, genetic, benefits affect female mate choice. Females can increase their own fitness when their offspring are more likely to survive (Hamilton & Zuk, 1982), bet‐hedge when selection favors genetically diverse offspring (Fedorka & Mousseau, 2002; Uller & Olsson, 2008), potentially may “trade‐up” by mating with a perceived higher quality male than their first mate (Pitcher, Neff, Rodd, & Rowe, 2003), thereby acquiring “good genes” for their offspring (Yasui, 1997), and mating multiply may allow for sperm competition, increasing the likelihood of high quality offspring (Eberhard, 1996; Jennions & Petrie, 2000). Females may mate with additional “better” males to increase offspring fitness (Pitcher et al., 2003; Zbinden, Largiader, Leippert, Margaritoulis, & Arlettaz, 2007) or could mate with multiple individuals to ensure sufficient sperm supplies and genetic diversity. In some cases, females can manipulate successful mating events such that mating may not lead to fertilization, making observed mating behavior misleading. Such cryptic female choice mechanisms can include morphological or chemical barriers to successful fertilization and can occur after insemination or even after fertilization (Brennan et al., 2007; Fedina & Lewis, 2004; Reeder, 2003).

Females also suffer costs imposed by mating multiply. Mating costs energy that might otherwise be allocated (Jennions & Petrie, 2000; Watson, Stallmann, & Arnqvist, 1998) may increase the risk of predation (Margaritoulis & Touliatou, 2011), physical injury (Evans & Magurran, 2000; Le Galliard, Fitze, Ferrière, & Clobert, 2005; Rowe, Arnqvist, Sih, & Krupa, 1994), and risk of infection (Forbes, 2014; Johns, Henshaw, Jennions, & Head, 2019; Roberts, Evison, Baer, & Hughes, 2015; Wardlaw & Agrawal, 2018). In courtship and attempts to access females, males may injure females (Ramm et al., 2015; Reinhardt, Anthes, & Lange, 2015) and mate avoidance can be costly (Hays et al., 2002). The sum of costs of multiple mating can decrease a female's lifespan and reduce the value of mating with multiple individuals, especially if the first mating provides enough sperm for fertilization (Pearse & Avise, 2001). Many species do mate multiply (Kokko & Mappes, 2013; Lee & Hays, 2004; Lewis, FitzSimmons, Jamerlan, Buchan, & Grigg, 2013; Liu et al., 2015), despite costs to females, suggesting that benefits outweigh costs or that males can coerce females (Eberhard, 1996; Yasui, 1998).

For each individual, benefits and costs will have differing values, but the value from the sum of these costs and benefits is expected to predict the likelihood of multiple mating. When costs and benefits are similar and the sum of fitness effects is zero (i.e., 1:1, Figure 1), females are expected to have little preference and mating patterns may vary with species (Kirkpatrick, 1982; Tokarz, 1995). When the costs are low, and the benefits are high, the likelihood of mating multiply increases. If the costs are high and benefits are low, females may evade or reject males, decreasing the likelihood of multiple mating (Fisher, Double, Blomberg, Jennions, & Cockburn, 2006; Shine, Wall, Langkilde, & Mason, 2005). When both costs and benefits are high, females may rely on cryptic choice (Eberhard, 1996).

**Figure 1:**
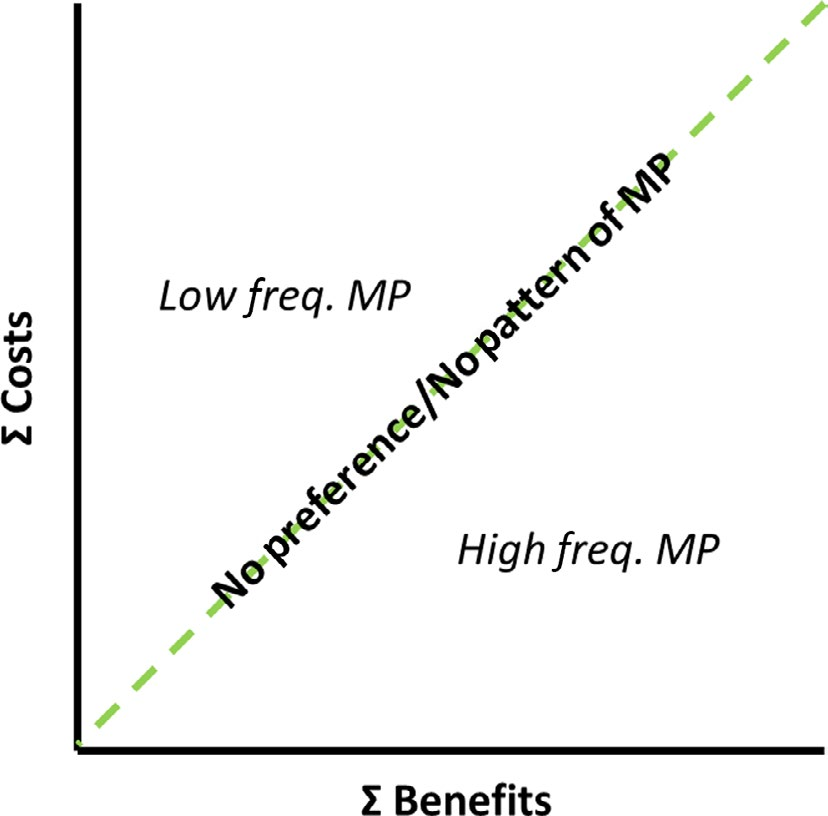
Model of potential costs and benefits of multiple mating that can result in patterns of multiple paternity (MP). For females, if the sum of benefits equals the sum of costs of mating (dashed line), then there will be no net fitness effect from mating with multiple males, making it difficult to predict any pattern of multiple paternity (MP), especially when both benefits and costs are small. As the sum of costs increases more than the benefits, multiple paternity frequency drops. As the sum of benefits increases more than the costs, multiple paternity increases

Though populations may tend toward a 1:1 sex ratio when both sexes are equally costly to produce (Bull & Charnov, 1988; Fisher, 1930), sex ratio biases can occur and persist, limiting the number of available mates. For many species of reptiles, an individual's sex is determined by environmental factors after fertilization (Bachtrog et al., 2014; Bull, 1980; Janzen & Krenz, 2004), and in these species, extreme environmental conditions can cause sex ratio bias (Hays, Mazaris, Schofield, & Laloë, 2017; Reneker & Kamel, 2016). In marine turtles, warmer temperatures during nest incubation produce more females and cooler temperatures produce more males (Mrosovsky & Yntema, 1980). Studies of hatchlings (Mrosovsky & Provancha, 1992; Wyneken & Lolavar, 2015) and juveniles (Jensen et al., 2018) have identified populations with strongly female‐biased sex ratios that may limit female choice when they become adults (Gaos et al., 2018).

For marine turtles and other species without parental care, there are no obvious direct benefits of multiple mating, yet, several studies show that marine turtles mate multiply within a breeding season (Crim et al., 2002; Fitzsimmons, 1998; Moore & Ball, 2002). Lasala, Hughes, and Wyneken (2018) examined the paternity of a small nesting assemblage of loggerhead sea turtles (*Caretta caretta*, Linnæus, 1758) in southwestern Florida and showed that most females mated multiply and that the breeding sex ratio (BSR) was male‐biased.

The goal of this study was to reveal paternity patterns between subsequent clutches to identify whether female preference determines the likelihood of multiple mating. We integrated genotypes and size class data from Lasala et al. (2018; data available on Dryad digital repository: https://doi.org/10.5061/dryad.q2kf0) to assess female choice in this population. We hypothesized that, if the benefits outweigh the costs (Figure 1), females should mate multiply both early and throughout the nesting season (i.e., between same‐year nesting events: remating). Finally, we examined potential indirect benefits that might encourage males or females to have preferential mating with multiple individuals.

## 2 MATERIALS AND METHODS

Loggerhead turtles tagged at Sanibel Island, Florida, USA (26.47058, −82.17347), often return to nest within the same season (renesting) (Lasala et al., 2018; LeBuff & Beatty, 1971), but the frequency of renesting is unknown. Flipper tag and satellite tag data for nearby nesting sites show that, in this region, females lay an average of 3.9–5.4 nests per nesting season (Addison, 1996; Tucker, 2010). This assemblage of turtles also nests at other suitable nesting sites to the north and south of Sanibel. With the aid of Sanibel Captiva Conservation Foundation (SCCF) staff, nesting turtles were identified at night by their flipper tags and were sampled for genetics from May to July of 2016. Tag numbers were used to determine whether individual turtles had nested at Sanibel previously. When nesting loggerhead turtles were found, flipper tags were checked or applied and data were collected following protocols by Lasala et al. (2018). Standard measurements of female body size were taken: curved carapace length (CCL), curved carapace width (CCW), straight carapace length (SCL) and straight carapace width (SCW). Blood was taken (<1 ml) using vacutainers with sodium heparin and frozen at −80°C until analysis; a small skin sample, collected using a sterile scalpel blade, was stored in 70% ethanol as a backup sample until analysis. Any untagged turtle encountered received two metal flipper tags and a PIT tag for identification. When a turtle was identified as renesting, her nests were included in this study, her genotype was identified using exclusion analysis, and her original and subsequent nests were marked for future sampling.

Approximately 45 days after egg deposition, restraining cages were placed over the nest chambers to prevent predation and ensure hatchlings could be obtained upon emergence. Up to 20 randomly selected hatchlings were sampled per clutch, ~1/5 of the clutch. The sample size was selected because if multiple paternity cannot be identified with 20 hatchlings, it is statistically unlikely present. Hatchling body measurements (SCL) were taken using vernier calipers. Blood samples were taken (100 µl per turtle) from the external jugular veins using heparinized 26G, ½ inch allergy needles (BD PrecisionGlide® Needles). A skin sample (~1 mm × 4 mm) from the trailing edge of one of the flippers was taken using a sterile scalpel blade. Blood and skin samples were treated as for those from nesting females. All hatchlings from each nest were released the night of emergence. Per SCCF guidelines, three days following hatchling emergence, the contents of the nest were inventoried to document nest success metrics. Emergence success (*E*) is quantified:

(1) $$E=\frac{\left(H-\left(L+D\right)\right)}{T}$$

where *H* is the number of hatched eggs,* T* is the total number of eggs, *L* is the number of live hatchlings in the nest, and *D* is the number of dead hatchlings in the nest. Emergence success was quantified to identify the number of hatchlings that successfully leave the nest without assistance. It is a proxy of hatchling quality and is more informative to individuals entering the population than hatch success (the number of hatchlings that hatch from their eggs).

Sample processing followed protocols in Lasala et al. (2018). PCR amplifications were carried out using primers for seven nuclear microsatellite loci following published protocols: CcP7E05, CcP2F11, CcP7D04, CcP5H07, CcP7C06, CcP7B07, and CcP8D06 (Shamblin et al., 2009). PCR products were multiplexed together and analyzed with a GeneScan500 fluorescent size standard (Applied Biosystems) using an ABI 3730 DNA Analyzer. Positive and negative controls were run for every extraction and PCR amplification to identify potential contamination. Alleles were identified using the program Geneious R10 (Biomatters Inc) and visual verification. Loci were checked for allelic dropout, stutter, and null alleles using the program MicroChecker 2.2.3 (Van Oosterhout, Hutchinson, Wills, & Shipley, 2004). Maternal genotypes from Sanibel collected 2013–2015 (Lasala et al., 2018; data available on Dryad digital repository: https://doi.org/10.5061/dryad.q2kf0) were added to maternal samples from this study (2016) to create a more accurate gene frequency estimation of the population, and a subset of all nesting females was examined for genotyping error rate using Pedant software (Johnson & Haydon, 2007).

Paternity was assessed through exclusion analysis using the program COLONY 2.0 (Jones & Wang, 2010). COLONY identifies paternal alleles from hatchling genotypes when the mother's genotype is known. It also can identify both parents if neither parent is known using population allelic frequency data. Multiple paternity within a clutch was determined by the presence of more than two paternal alleles over at least two loci, conservatively allowing for a mutation at one locus (Yue & Chang, 2010). We calculated the breeding sex ratio for 2016 and identified the minimum number of individuals contributing to clutches. Identified male genotypes were compared to identify if males were mating with multiple females (males ID'd in multiple nests) and if females were remating between nesting events (statistically different male contributions between subsequent nests).

The program GenAlEx (Peakall & Smouse, 2012) was used to determine (a) the observed and expected heterozygosity and deviations from Hardy–Weinberg equilibrium of the maternal genotypes, (b) the probability of identity (PI), and (c) the probability of exclusion (PE). PI and PE provide estimates that the loci used are a good representation of the genotype. PI is the likelihood that two samples will have the same genotype based upon the estimated allelic frequencies at that locus and when all loci are combined. PE is the proportion of the population that has a genotype that contains at least one allele not present in the mixed profile. PE can depend on how many parents are known, so we include PE_2_ (one parent known) and PE_3_ (if no parents are known) for all markers.

Subsequent clutches that had multiple paternity were compared to assess the paternal contributions (Sørensen similarity index: QS):

(2) $$\text{QS}=\left(\frac{2C}{A+B}\right)*100\%$$

where *A* and *B* are the number of fathers in each clutch, and *C* is the number of fathers shared between clutches (adapted from Sorensen, 1948 and Figgener, Chacón‐Chaverri, Jensen, & Feldhaar, 2016). For multiply sired clutches, the probability (*p_f_*) that males identified in previous clutches would *not* appear in subsequent clutches due to chance (i.e., random mating) was identified using:

(3) $$p_{f}={\left(1-f\right)}^{n}$$

where *f* is the proportion of the first clutch sired by the first male and *n* is the number of hatchlings sampled.

All analyses were run using Statistical Analysis System (SAS). Student's *t* tests were conducted to identify whether female size (as defined by CCL, CCW and a ½ ellipsoid surface area) differed between single and multiple fathered nests. Ellipsoid surface area (SA) is defined as:

(4) $$\text{SA}=4\pi {\left(\frac{a^{1.6}b^{1.6}+a^{1.6}c^{1.6}+b^{1.6}c^{1.6}}{3}\right)}^{1/1.6}$$

where *a*,* b*, and *c* are the principal semi‐axes: *a* = ½ SCL, *b* = ½ SCW, and *c* is derived from the CCL length (1/2 perimeter of an ellipse). We did not take ventral measurements, so the total surface area is divided by 2. Student's *t* test was conducted to assess whether hatchling size and emergence success differed between the initial and secondary clutches and whether emergence success differed between single and multiple paternity nests. Chi‐square tests were conducted to identify whether hatchling size differed between single and multiple paternity clutches. *F* tests were conducted to determine whether there was a difference in variance of hatchling size between primary and secondary clutches and between single and multiple paternity clutches. Because just four turtles laid tertiary nests at Sanibel, the third clutches were excluded from these analyses. Any female whose primary clutch was inundated with water was removed from these analyses of nest success or hatchling size as these clutches typically experience partial or complete mortality.

Using data from Lasala et al. (2018), we conducted linear regression analyses to predict whether the number of successful fathers was dependent on the body size (CCL, CCW, and the ½ ellipsoid surface area) of the nesting females. A linear regression was run to predict whether hatchling size (SCL) was dependent on female size (CCL/CCW). Female size values were divided to decrease the variation due to shell curvature that would not be present in the hatchlings. Finally, linear regressions were run to predict whether hatchling size was dependent on the date the nest was laid and whether size was dependent on incubation duration.

## 3 RESULTS

### 3.1 Nesting

Sixteen of the turtles that SCCF encountered in 2016 laid multiple nests on Sanibel. The shortest time between nesting sightings was 10 days and the longest was 38 days (Table 1). Thirty‐six clutches were sampled and analyzed (587 hatchlings). In some cases, <20 hatchlings hatched or emerged from their nests.

**Table 1:** Table identifying number of fathers and days between female encounters

Female ID	1° Clutch# fathers	2° Clutch# fathers	Tertiary Clutch # fathers	Days between counters
SSA714	1	1	1	24, 26
LLZ512	1	1		23
LLZ588	3	2		25
LLZ526	1	1	1	11, 12
LLZ650	1	1	1	25, 13
LLZ506	1	1		10
LLZ670	1	1		11
LLZ678	1	1		23
LLZ692	1	1		21
LLZ912	1	1		21
LLZ918	1	1		12
LLZ591	5	5		27
LLZ930	1	1	1	11, 10
LLZ948	3	2		13
LLZ963	2	2		11
LLZ640	1	1		12

Note

Four turtles laid more than two clutches. Days between observed nesting events are also shown. Females highlighted in gray mated with multiple males. It is likely that the turtle whose interesting interval is 20–25 days laid an intervening clutch elsewhere. The species typical interesting period is 10–13 days (mode = 11).

### 3.2 Genetics

Combining the seven loci resulted in a PI of 5.0 × 10^−13^, and a PE_2_ of 0.999 (when one parent was known) and PE_3_ of 1.000 (when neither parent was known). Microchecker did not detect evidence of scoring error due to stutter, allele dropout, or null alleles. The genotyping error rate from 5.1% of the nesting females from 2013 to 2016 was 2.1%. The number of alleles in the population ranged from 14 to 25 across loci (including novel alleles detected in 2016); there were no significant deviations from Hardy–Weinberg equilibrium. Females in this population mate freely within reproductive seasons (panmixis; Lasala et al., 2018).

### 3.3 Multiple paternity patterns

**Figure 2:**
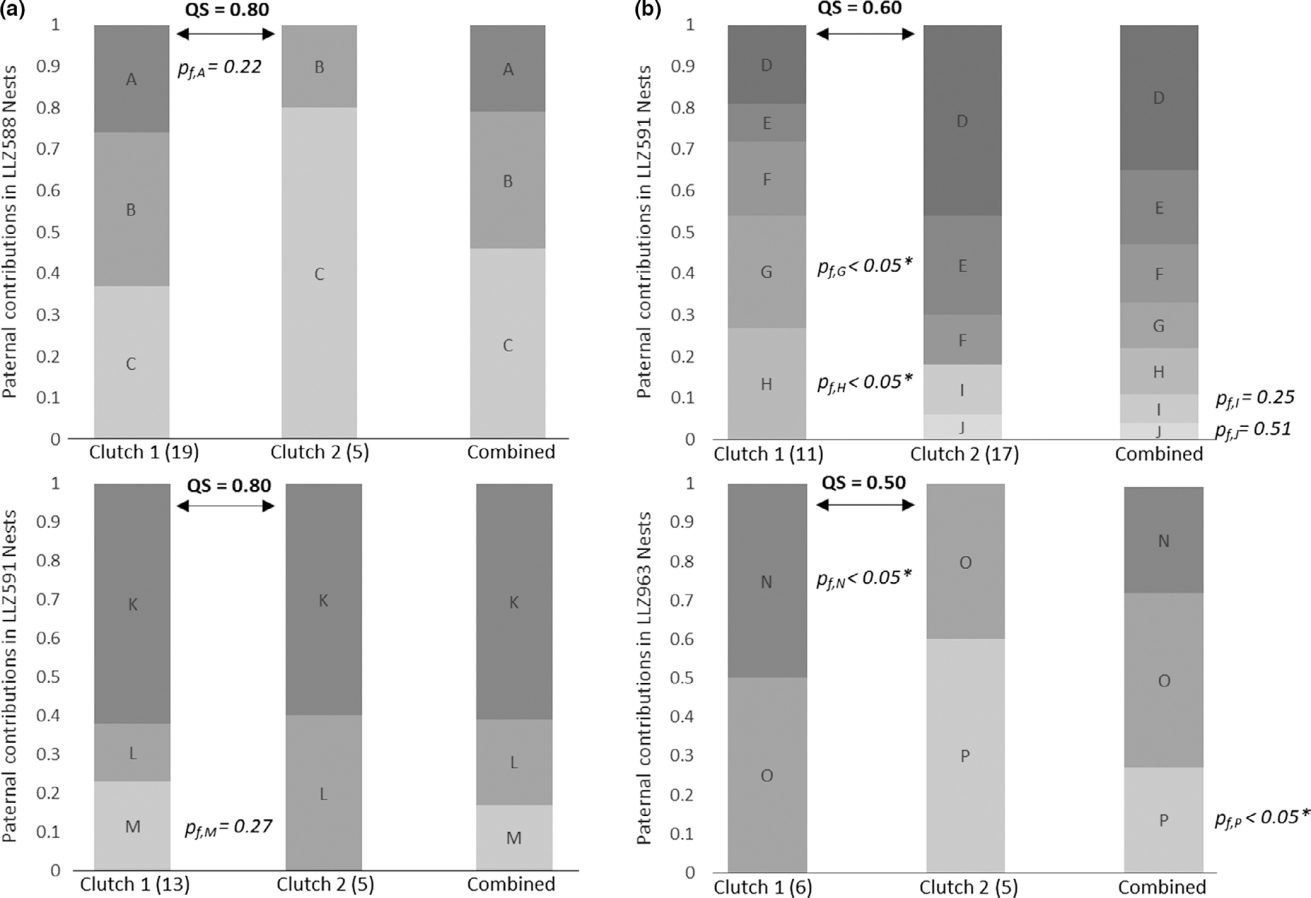
Proportions of paternal contributions between subsequent clutches with multiple paternity. Each bar represents the proportion of the clutch each father sired, the number of hatchlings analyzed is in parentheses after each clutch number (*n*). The Sørensen similarity index (QS) indicates how similar the composition of fathers is between clutches. In cases where fathers are found in one clutch but not the other, the probability that their contributions would not be observed through random chance is given by Pf. Probabilities noted with an * are highly unlikely to have occurred by chance alone (refer to Equation 3). The clutches in Group a show patterns of storage then depletion of sperm likely due to chance. Clutches in Group b show patterns that are likely due to mating between nesting events

Twelve females produced 28 clutches that were all singly sired, where the father identified in the first clutch's offspring was also the only father found in subsequent clutches. Four females produced eight clutches sired by multiple males (22% of all clutches examined, Table 1). At least one male identified in the first clutch was also identified in the second. In two cases, new paternal genotypes were present in the second clutch. For example, in female LLZ963's first clutch, there were two paternal contributions designated here as males *N* (50%) and *O* (50%), and in her second clutch males, *O* (40%) and* P* (60%) were the fathers (Figure 2). No females laid clutches where the male genotype was also identified in the clutch of a different female.

The breeding sex ratio for 2016 was calculated as 1.46 males per female. In 2016, 634 loggerhead nests were laid on Sanibel, if females in the region lay 3.9–5.4 nests per season (Addison, 1996; Tucker, 2010), we calculate that there must be at least 117–163 females represented by these nests. Consequently, we estimated there were 171–238 males contributing to clutches in Sanibel in 2016. For comparison, if breeding sex ratio was calculated based on the first clutches alone, it shifts slightly to 1.56 males per female. Comparison of the two breeding sex ratio estimates found no difference (Fisher's exact test *p* = .800).

Figure 2 illustrates the paternal contributions for the four females' clutches that were sired by multiple males. Those clutches ranged in similarity (QS) between 50% and 80%. Contingency tests did not indicate a statistically significant difference in proportions of hatchlings fathered by each male between subsequent clutches, with one exception: female LLZ591 (Figure 2b). In her first clutch, fathers *G* and *H* were identified but neither was found in the second clutch and in their place were fathers *I* and *J* (χ^2^ = 5.844, *df* = 6, *p* = .015). To assess probable explanations for this change (nonappearance of fathers in subsequent clutches), we calculated *p_f_* as if stored sperm are well‐mixed; then, the first clutch is the best estimate of relative sperm contributions, then the nonappearance of fathers *G* and *H* in subsequent clutches is likely due to chance or sperm depletion (*p_f_*_,_ *_G_* = .22 and *p_f_*_,_ *_H_* = .27). We then calculated *p_f_* to determine whether the nonappearance of fathers *G*, *H*, and *N* is unlikely due to chance alone (*p_f,G,H,N_* < .05; Group a Figure 2). If the relative contributions of the fathers are best‐estimated from the combined genotypes of both clutches, then the appearance of fathers *I* and *J* is likely due to chance (sperm mixing or remating; *p_f_*_,_ *_I_* = .25 and *p_f_*_,_ *_J_* = .51), but the appearance of father *P* is unlikely due to chance and likely a result of remating (*p_f,P_* < .05; Group b Figure 2).

### 3.4 Measurements

Female measurements (CCL, CCW, SCL, SCW) were distributed normally (Shapiro–Wilk, *p* = .57, .93, .56, .63, respectively) and the residuals were all homogenous. Between single and multiple paternity nesters, female length and width were not significantly different (CCL: *t*_13_ = 0.937, *p* = .183; CCW: *t*_13_ = 0.806, *p* = .217), but single paternity nesters had a significantly larger surface area (*t*_12_ = 2.177, *p* = .025) (Table 2a). Hatchling straight carapace length (SCL) was also identified to be normally distributed (Shapiro–Wilk, *p* = .52) and their residuals were homogenous. There was no difference in hatchling size or variance of hatchling size (Table 2b) between primary and secondary clutches (SCL: *t*_26_ = −0.31, *p* = .379, Var: *F*_290, 230_ = 1.19, *p* = .074) and no difference in hatchling size due to the number of fathers ($\chi_{26}^{2}$ = 0.006, *p* = .939). There was a significant difference in variance of hatchling size between single (3.18) and multiple paternity (2.47) clutches (*F*_436, 143_ = 1.29, *p* = .036).

**Table 2:** Descriptive measurements of females (a) and hatchlings (b)

(a)		
	Single paternity	Multiple paternity
SCL	97.2 cm (7.1)	93.5 cm (2.3)
SCW	88.1 cm (5.3)	85.7 cm (2.9)
½ Ellipsoid surface area	5,931.6 cm^3^ (641.4)	5,007.9 cm^3^ (716.7)

(b)		
	1st Nest	2nd Nest
SCL	42.69 (1.6)	43.28 (1.7)
Variance	2.57	3.08

Note

Straight carapace length (SCL), straight carapace width (SCW). Values within parentheses are the standard error. Females who laid single paternity nests were larger (surface area was statistically significant) than females who laid multiple paternity nests. Hatchlings were larger and more variable (significant) in subsequent nests than primary nests.

Comparison of clutch success found no statistical difference in emergence success between primary (0.61) versus secondary clutches (0.59) (*t*_28_ = 0.15, *p* = .441) and single (0.61) versus multiple paternity clutches (0.57) (*t*_10_ = 0.259, *p* = .400).

**Figure 3:**
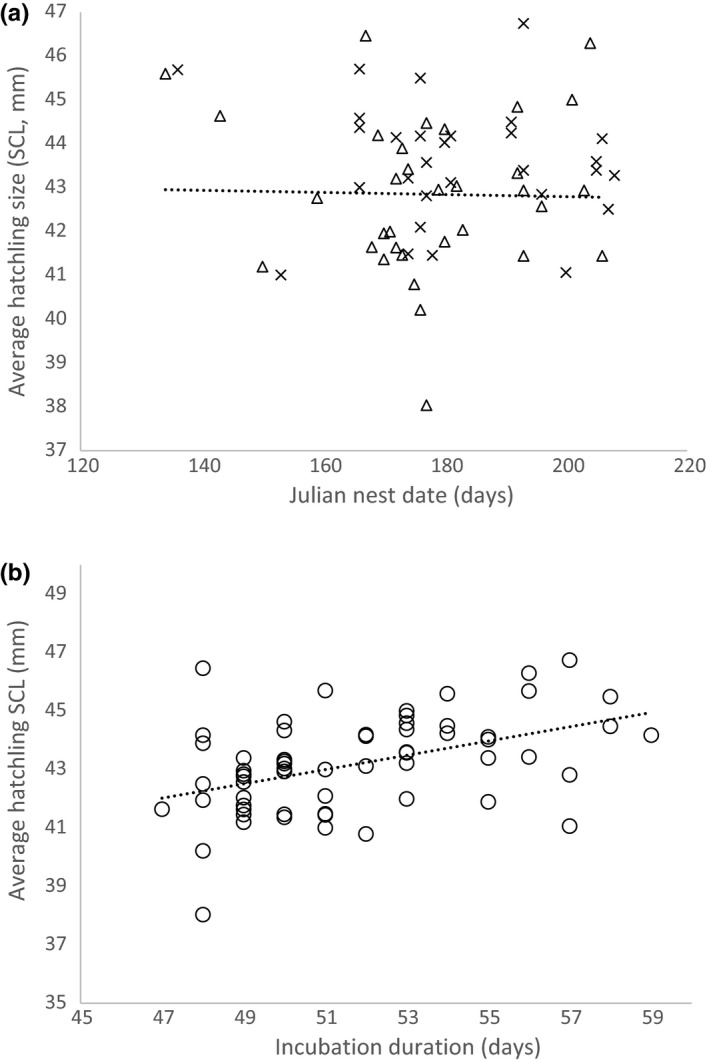
(a) Julian nest date as a predictor of hatchling size (SCL, mm). Nesting dates from 2013 to 2016 in relation to hatchling size were not a significant predictor of hatchling size for loggerheads in Sanibel. Julian date 120 is May 1st. Single paternity nests shown by Δ and multiple paternity nests by *X*. Regression equation: *y* = −0.004*x* + 43.838, *R* ^2^ = .001. (b) Incubation duration of nests in relation to hatchling size (SCL, mm). Nest incubation duration from 2013–2016 shows a significant increase in hatchling size as incubation duration increases. Regression equation: *y* = 0.244*x* + 30.567, *R* ^2^ = .201

Female length and width were not significant predictors of the number of fathers (CCL: *F*_1,58_ = 0.347, *p* = .558, *R* ^2^ of .005; CCW: *F*_1,58_ = 0.004, *p* = .948, *R* ^2^ < .001). The ratio of CCL/CCW was not a significant predictor of hatchling size (*F*_1,61_ = 0.019, *p* = .892, *R* ^2^ < .001). Finally, across all years, the Julian date of the nest deposition was not a significant predictor of hatchling size (*F*_1,61_ = 0.086, *p* = .770, *R* ^2^ = .001, Figure 3a), but incubation duration was a significant predictor of hatchling size, longer incubation durations resulted in larger hatchlings (*F*_1,61_ = 15.305, *p* < .001, *R* ^2^ = .201, Figure 3b).

## 4 DISCUSSION

Several studies show that sea turtles mate multiply but there is no consensus on why they do so (Harry & Briscoe, 1988; Kichler, 1999; Moore & Ball, 2002; Stewart & Dutton, 2014; Tedeschi et al., 2015; Wright et al., 2012). The frequency of multiple mating increases as the number of nesting females increases for olive Ridley sea turtles (*Lepidochelys olivacea*, Jensen, Abreuu‐Grobois, Frydenberg, & Loeschcke, 2006), yet similar trends are lacking for *Caretta*. When multiple mating is observed visually (Booth & Peters, 1972; Kawazu, Okabe, & Kobayashi, 2017), there is no certainty of fertilization, yet it is hypothesized that females may mate multiply to ensure fertilization (Uller & Olsson, 2008) resulting in multiple paternity within nests.

The frequency of multiple paternity from this study was 22% (8 of 36 nests), which is lower than previously reported for this nesting beach (67%, 34 of 51 nests) (Lasala et al., 2018). The simplest explanation for the 12 females with singly fathered nests is that they successfully mated once and stored sufficient sperm to fertilize all the eggs in the multiple clutches observed. This result suggests that the “fertilization insurance” hypothesis is not supported compellingly. Previous studies examining marine turtles show that females can store viable sperm for the full nesting season (Sakaoka, Yoshii, Okamoto, Sakai, & Nagasawa, 2011) and that nests laid weeks apart had the same paternal contributions with sufficient sperm quantity (Fitzsimmons, 1998; Joseph, Chong, & Shaw, 2017; Phillips et al., 2013, this study). Nielsen (2010) reasoned that unless females are remating with the same males that sired their first clutches sperm storage is the most likely explanation. In a large breeding population, it is unlikely that females mate with the same individual males between breeding seasons (Sakaoka, Sakai, Yoshii, Okamoto, & Nagasawa, 2013). However, there is observational evidence that during the internesting period, female leatherback turtles (*Dermochelys coriacea* L.) attempt to avoid males and discourage mating attempts (Reina, Abernathy, Marshall, & Spotila, 2005), supporting the idea that most females complete mating prior to their first nesting. In our study, all clutches had male genotypes that persisted from the first clutch to subsequent clutches (including one of 50 days past the first observed clutch). Males appear to complete their breeding season before all females complete nesting (Wibbels, Owens, Amoss, & Witzell, 1987) and depart for foraging grounds (Arendt et al., 2012; Lee, Schofield, Haughey, Mazaris, & Hays, 2017). Given the dispersion of loggerhead turtles and the lack of evidence of pair bonds, it is improbable that females who laid singly sired nests would seek out the same specific males to replenish their sperm storage.

In clutches from the four females that successfully mated with multiple males, the paternal contributions differed between sequential clutches. Four nonexclusive explanations are possible: (a) Stored sperm is not well‐mixed, (b) some paternal contributions are depleted over time, (c) females mated between nesting events effectively shifting the proportions of each male's sperm, and (d) there may be differences in the success of different males' sperm in fertilization. In Figure 2a, the nonappearance of two paternal genotypes (*A* upper left, *M* in lower left) in the respective second clutch is more likely due to random chance or sperm depletion. Unfortunately, the statistical power of this study was limited by environmental impacts on incubation. In 2016, record high temperatures occurred during much of the summer incubation period (NOAA, 2017) and overall hatching success was reduced due to heat‐related embryo mortality. Consequently, few hatchlings emerged from the second clutches and fathers *A* and *M* might have been missed by chance. Alternatively, unless those fathers' eggs were more prone to failure, and we have no evidence for such, their sperm contributions were depleted before the second clutches. In Figure 2b, male genotypes were identified in primary clutches, but were replaced with new genotypes in subsequent clutches (e.g., *G*/*H* →*I*/*J*, and* N* →*P*). The loss of fathers *G*, *H*, and *N* are unlikely due to chance because of their high proportions in the first clutch; here, the most likely explanation is that these females mated between nesting events. The presence of *I* and *J* only in the later clutch could be due to random chance detection, but the proportion of father *P* in the second nest could only be due to a new mating event.

New paternal contributions after the first nest were identified in loggerhead clutches in Australia (Harry & Briscoe, 1988), leatherback clutches in Costa Rica (Figgener et al., 2016), and green turtle (*Chelonia mydas* L.) clutches in Mexico (Chassin‐Noria, Macip‐Ríos, Dutton, & Oyama, 2017). In these studies, and in our study, the incidence of mating between nests appears to be low, suggesting that the benefits of remating may to be low.

If females were mating with multiple males to increase opportunities for and benefits from “good” genes, we would expect to see higher emergence success and/or larger (and presumably, more robust) hatchlings in multiple paternity clutches. There were no differences between primary versus secondary clutches or between single versus multiple paternity clutches. Surprisingly, the metrics of hatchlings from single paternity clutches were more variable than multiple paternity clutches and those from primary clutches were more homogenous than secondary clutches. This latter result might be due to remating; however, the nest environment, particularly moisture content, can also affect hatchling sizes (Erb, Lolavar, & Wyneken, 2018; McGehee, 1990). The year in which this study was conducted was hotter than normal (NASA, 2017).

As the nesting season progresses, loggerhead clutch sizes decrease and energy for eggs is depleted (Ehrhart & Witherington, 1987; Frazer & Richardson, 1985). Loggerhead females fast during the nesting season (Deem et al., 2009). In general, larger hatchlings tend to emerge from nests with longer incubation durations. In our study, hatchling size differences were not detectable over the course of the season, suggesting that multiple factors within the nest (e.g., water availability, gas exchange, yolk utilization) may mask genetic influences from the parents. In this southwestern Florida nesting population, indirect benefits of more robust hatchling size were not supported and provided no insight into the variation of the number of fathers.

The relationship between multiple paternity and female size varies among studies. In our study, multiply‐mated females were smaller than singly mated dams. This is consistent with loggerheads nesting on the Florida panhandle (Nielsen, 2010) (both are Gulf of Mexico nesting sites although >500 km apart). The four females with new fathers in subsequent clutches were smaller and possibly younger than those that mated with one male. It is possible that smaller females or those breeding for the first time may be unable to reject persistent or aggressive males and hence their nests would be prone to multiple paternity. Our results differ from those for loggerheads in the Mediterranean (Zbinden et al., 2007) and Georgia, USA (Lasala, Harrison, Williams, & Rostal, 2013), in which females that mated multiply were longer than females that mated singly. Hypothetically, males along the Gulf Coast of Florida, USA, might prefer smaller females or mate choice might differ among subpopulations. However, a likely hypothesis to explain these results is that larger, potentially older, females might be more experienced at rejecting additional males.

The estimated breeding sex ratio for this population is male‐biased and a high number of male genotypes were identified. It is unlikely that females are constrained from multiple mating by scarcity of potential mates. The strong female‐bias in primary sex ratios indicates that current breeding sex ratios are affected by factors other than hatchling sex ratios. Several factors may explain the breeding sex ratio including the potential that males mate annually (Limpus, 1993; Wibbels, Owens, Limpus, Reed, & Amoss, 1990; Wright et al., 2013), male turtles concentrating near nesting beaches increases encounter rates (Lasala et al., 2013; Lee et al., 2017), and females mate with males outside of the nesting area (Lasala et al., 2018). Should the breeding sex ratio become female‐biased in the future, then multiple mating would be predicted to be less common than it is today and males fathering more than one female's nest may be detected.

We conclude that the data are most consistent of there being little benefit and little cost to multiple mating by females. It is reasonable to hypothesize that larger and more experienced females may be more effective in controlling their numbers of mates than smaller, neophyte nesters. Together our data are consistent with the hypotheses that females may mate on their way to nesting beaches as well as near their nesting beach and store enough sperm for their entire breeding season (Lasala et al., 2018). While loggerhead females may mate between nesting events, that behavior appears to be relatively rare.

## CONFLICT OF INTEREST

None declared.

## AUTHOR CONTRIBUTIONS

Dr. Jacob Lasala conceived the project, collected/analyzed data, and took leading role in writing manuscript. Drs. Colin Hughes and Jeanette Wyneken helped to conceive the project, provided logistical support, and assisted in writing the manuscript.

### Open Research Badges

This article has earned an Open Data Badge for making publicly available the digitally‐shareable data necessary to reproduce the reported results. The data is available at https://doi.org/10.5061/dryad.q2kf0; https://doi.org/10.5061/dryad.t38b3k2

## Data Availability

Previous genotypes from Lasala et al., 2018 are available on Dryad here: https://doi.org/10.5061/dryad.q2kf0. Genotypes of individuals and morphometric data are available on Dryad here: https://doi.org/10.5061/dryad.t38b3k2
