# Supplementary material for: Female loggerhead sea turtles (Caretta caretta L.) rarely remate during nesting season
Source: Ecol Evol. 2019 Dec 20;10(1):163–74. doi: 10.1002/ece3.5869 (PMC6972835; doi:10.1002/ece3.5869)
Supplement: Supplementary file 1 [file ECE3-10-163-s001.docx]

**SUPPLEMENTAL INFORMATION**

Example for using equation #3:

$$P_{f}={(1-f)}^{n} (3)$$

where *f* is the proportion of the first clutch sired by the 1^st^ Male and *n* is the number of hatchlings sampled.

For example: If in a theoretical Clutch 1, the 1^st^ Male was the father for 25% of the hatchlings and the 2^nd^ Male 75%. When comparing to a theoretical Clutch 2 (n =10), because each offspring’s parentage is independent, the probability the 1^st^ Male would *not* be identified in at least one offspring is: (1-0.25)^10^ = 0.056. In this example, it is highly likely that the 1^st^ Male would be found in Clutch 2.
